# A Deep Insight in the Antioxidant Property of Carnosic Acid: From Computational Study to Experimental Analysis

**DOI:** 10.3390/foods10102279

**Published:** 2021-09-26

**Authors:** Jing Wei, Qian Liang, Yuxin Guo, Weimin Zhang, Long Wu

**Affiliations:** 1 Engineering Research Center of Utilization of Tropical Polysaccharide Resources (Ministry of Education), College of Food Sciences & Engineering, Hainan University, 58 People Road, Haikou 570228, China; weijing0128@126.com (J.W.); lq9507252021@163.com (Q.L.); yuxinguo202109@163.com (Y.G.); 995050@hainanu.edu.cn (L.W.); 2 Key Laboratory of Tropical Fruits and Vegetables Quality and Safety for State Market Regulation, Hainan Institute for Food Control, 285 Nanhai Road, Haikou 570314, China; 3 Hubei Key Laboratory of Industrial Microbiology, Key Laboratory of Fermentation Engineering (Ministry of Education), Hubei University of Technology, Wuhan 430068, China

**Keywords:** carnosic acid, oils, thermal oxidation stability, inhibition mechanism, density functional theory

## Abstract

Since the deep cause for the anti-oxidation of carnosic acid (CA) against oleic acid (OA) remains unclear, we focused on exploring the CA inhibition mechanism via a combined experimental and computational study. Atomic charge, total molecular energy, phenolic hydroxyl bond dissociation enthalpy (BDE), the highest occupied molecular orbital (HOMO), and the lowest unoccupied orbital (LUMO) energy were first discussed by the B3LYP/6-31G (d,p) level, a density functional method. A one-step hydrogen atom transfer (HAT) was proposed for the anti-oxidation of CA towards OA, and the Rancimat method was carried out for analyzing the thermal oxidation stability. The results indicate that the two phenolic hydroxyl groups located at C_7_(O_15_) and C_8_(O_18_) of CA exert the highest activity, and the chemical reaction heat is minimal when HAT occurs. Consequently, the activity of C_7_(O_15_) (303.27 kJ/mol) is slightly lower than that of C_8_(O_18_) (295.63 kJ/mol), while the dissociation enthalpy of phenol hydroxyl groups is much lower than those of α-CH_2_ bond of OA (C_8_, 353.92 kJ/mol; C_11_, 353.72 kJ/mol). Rancimat method and non-isothermal differential scanning calorimetry (DSC) demonstrate that CA outcompetes tertiary butylhydroquinone (TBHQ), a synthetic food grade antioxidant, both in prolonging the oxidation induction period and reducing the reaction rate of OA. The E_a_ (apparent activation energies of reaction) of OA, TBHQ + OA, and CA + OA were 50.59, 57.32 and 66.29 kJ/mol, revealing that CA could improve the E_a_ and thermal oxidation stability of OA.

## 1. Introduction

As a constant intake of trans fatty acids (TFAs) can pose detrimental effects on metabolic syndrome, heart disease and diabetes [1], much attention has been focused on obtaining safe and highly stable TFAs inhibitors from natural plant resources [2,3]. Carnosic acid (CA), one of the most thermally stable radical scavenging compounds with good solubility in fat, is the most abundant and active natural antioxidant among those present in rosemary [4,5]. Compared with the artificial antioxidants (PG, propyl gallate; BHT, butylated hydroxytoluene; BHA, butylated hydroxyanisole; Vitamin E and synthetic α-tocopherol), natural CA shows better performance on enhancing the oil stability [6,7]. Furthermore, CA plays an important part in pharmacological effects against cancer, cardiovascular diseases, diabetes, obesity, neuroinflammation and inflammatory disease [8,9]. Therefore, it is an interesting idea to explore CA with respect to the anti-oxidation mechanism.

It is known that oil oxidation is closely related to free radicals, and most antioxidants are free radical scavengers [10,11]. Density functional theory (DFT) has been widely adopted to study the anti-oxidation mechanism of different antioxidants, including carnosic acid, tea polyphenols, phenol carboxylic acids, and BHA, BHT, PG and TBHQ [12,13,14]. To date, two mechanisms of scavenging free radicals have mainly been reported for phenol antioxidants: direct hydrogen transfer (one-step hydrogen extraction reaction) and proton transfer [15,16]. CA has a diphenol diterpenoid structure with two hydroxyl groups on its benzene ring, with the capacity to be easily ionized and to act as excellent hydrogen donors [17]. This kind of phenol compound can easily form intramolecular hydrogen bonds and exert enhanced antioxidant activity in a preferred hydrogen extraction reaction mechanism, especially in nonpolar solvents like oil [18]. Meanwhile, polar solvents are beneficial to the charge separation, whereas hydrogen bonding is easily generated between polar solvents and antioxidants, which hinder the hydrogen extraction reaction [19,20]. Therefore, the fat-soluble CA has a high possibility to undergo the mechanism of direct hydrogen transfer. However, the mechanism for the antioxidant property of CA in oils remains unclear. Thus, it is of great importance to thoroughly evaluate the inhibition of lipid oxidation in oils.

Previous studies demonstrated that the autoxidation of oil begins with the separation of the α-H from the lipid C = C. Subsequently, the latter forms α-free radicals and reacts with oxygen to form peroxidized free radicals. A final free radical chain reaction then occurs to produce the first lipid oxidation product [21,22]. The lower the C-H bond energy is, the easier the hydrogen transfer will be. The hydrogen transfer can be indicated by bond length, bond order, bond energy and bond dissociation energies (BDE) [23,24]. Among these factors, BDE has widely been used to evaluate the stability of free radicals [25,26]. In oils, the lower BDE value of C-H bond reveals that free radicals are more stable, and therefore, oils are more susceptible to oxidation. In addition, the isomerization of unsaturated lipids entails α-free radicals that can induce the formation of conjugated isomers of lipids [27,28]. Various properties of CA have been investigated, including chemical structure, antioxidant activity, and oxidative stability [29,30,31], even though only a limited number of studies have reported the exact mechanisms involved. For example, Masuda et al. proposed an antioxidant mechanism of CA in which the oxidative coupling reaction is related with the peroxyl radical [17], however, the relevant theoretical simulations remain to be discussed. In addition, Liu et al. proposed an oxidation and isomerization pathway for CA via an o-quinone intermediate [32], however, their results have not yet been verified by any experimental work. 

We investigated the thermal oxidation and isomerization of OA by calculating the phenolic hydroxyl BDE and using DFT method, focusing on the hydrogen extraction reaction of α-H at C = C. Additionally, the phenolic hydroxyl BDE of CA was measured and compared with that of α-CH_2_ bond of OA. The differences between the two BDE values can help determine the mechanism of OA oxidation. The BDE of α-CH_2_ bond in OA calculated by DFT is a favored means for the discussion of OA isomerization. Among the commonly used artificial antioxidants (PG, BHT, BHA, TBHQ), TBHQ is the most stable and effective towards oils [33,34]. We explored the apparent activation energy (*E_a_*) of TBHQ and CA upon reaction with OA, which further demonstrated the superior antioxidative activity of CA. This research proposes an antioxidant mechanism of one-step hydrogen atom transfer for CA, based on computational study and traditional experiments, which revealed the detailed antioxidant process of CA.

## 2. Materials and Methods

### 2.1. Chemicals and Materials

Oleic acid (OA, 99%), 2,2-diphenyl-1-picrylhydrazyl (radial DPPH^•^, AR), 2,2-azinobis (3-ethyl-benzothiazoline-6-sulfonic acid) (radical ABTS^•+^), N-tert-butyl-alpha-phenylnitrone (PBN) and TBHQ (98%) were purchased from Sigma-Aldrich company(Shanghai, China); carnosic acid (CA, 98.6%) was purchased from Shanghai Yuanye Biological Technology Co., Ltd. (Shanghai, China); other chemicals were obtained from Sinopharm Chemical Reagent Co. Ltd. (Shanghai, China); Unless otherwise mentioned, the water used in the experiment was deionized and ultrafiltrated by Milli-Q device (Milli-Q, Millipore, Billerica, MA, USA, 18.2 MΩ resistivity). 

### 2.2. Determination of the Antioxidant Activity

The antioxidant activity of CA and TBHQ was determined by the previously described radial DPPH^•^ method with some modifications [35,36]. Typically, 4 mg radical DPPH^•^ was accurately weighed and dissolved in 95% methanol solution to 100 mL. Next, 1 mL CA and TBHQ of different concentrations (5, 10, 15, 20 and 25 μg/mL) were added into 3.0 mL DPPH solution, respectively. After standing still for 20 min at room temperature, the absorbances of mixtures (A_1_) were measured at 517 nm. The absorbance of 1.0 mL 95% ethanol solution mixed with 3.0 mL DPPH (A_0_) was determined as a negative control. The scavenging rate (SR) for radical DPPH^•^ was calculated according to the following equation:

SR_DPPH_^•^ (%) = [(A_0_ − A_1_)/A_0_] × 100% (1)

where A_0_ is the absorbance of the control samples and A_1_ is the absorbance of the samples.

Similarly, the radical ABTS^•+^ was also adopted to determine the antioxidant activity of CA and TBHQ [37]. In detail, 0.0384 g radical ABTS^•+^ and 0.134 g potassium persulfate were firstly dissolved, and the volumes were kept to 10 mL, respectively. After that, the two solutions were mixed together and allowed to stand still for 12 h to obtain ABTS^•+^ working solution. Prior to use, the absorbance of ABTS^•+^ solution was required to be 0.7000 ± 0.001 at 734 nm. Next, 0.3 mL CA and TBHQ with different concentrations (5, 10, 15, 20, 25 and 30 μg/mL) were reacted with 2.7 mL ABTS^•+^ working solution for 30 min, and the absorbance was measured at 734 nm. The scavenging rate (SR) for radical ABTS^•+^ was calculated according to the following equation:

SR_ABTS_^•+^ (%) = [(A_0_ − A_1_)/A_0_] × 100% (2)

where A_0_ is the absorbance of control (phosphate buffer) and A_1_ is the absorbance of samples.

### 2.3. The Exploration of CA Activity for Free Radicals Scavenge

The semiempirical quantum chemical methods (AM1) are commonly employed theoretical methods for the assessment of molecular properties [38,39,40]. In our research, AM1 and DFT methods were applied to study the activity of CA for scavenging free radicals (using Gaussian09 software). Firstly, AM1 was adopted to optimize the geometric structure of hydrogenated CA, by which the atomic charge, total molecular energy, phenolic hydroxyl bond dissociation enthalpy (BDE), the highest occupied molecular orbital (HOMO), and the lowest unoccupied orbital (LUMO) energy were discussed by the B3LYP/6-31G (d,p) level. Accordingly, the energy level difference between HOMO and LUMO (ΔE_(LUMO-HOMO)_) indicated the stability of the CA radical. The lower ΔE_(LUMO-HOMO)_ revealed lower stability and higher reaction activity. The BDE of hydroxyl groups was defined as BDE_O-H_ = H_CA radical_ + H_atom_ − H_CA_ (1), where H_CA radical_ represents CA free radical energy, H_atom_ represents hydrogen atom energy, and H_CA_ represents CA molecule energy. The BDE of C-H was defined as BDE_C-H_ = E_sr_ + E_hr_ − E_s_ (2), where E_sr_, E_hr,_ and E_s_ represents the energy of OA radical, α-H and OA, respectively. After that, the anti-oxidation activity of CA was analyzed in the view of HAT reaction, and the free radical scavenging mechanism of CA was further discussed.

### 2.4. Mechanism of α-Carbon Dehydrogenation of OA

Gaussian09 software was used to complete the molecular geometry optimization and frequency calculation at the B3LYP/6-31G (d,p) level. In turn, the geometric structure and enthalpy of OA, OA + CA, OA radical + CA before and after the HAT reaction were obtained. Additionally, we obtained the dissociation energy, bond length and bond order of the C-H bond, which can be used to clarify the mechanism of CA inhibiting the thermal oxidation reaction of OA.

### 2.5. Determination of Induction Period (IP)

The OA were exposed under a variety of temperatures (100, 110, 120 and 130 °C) in saturated air (20 L/h) using Rancimat (743 model, Metrohm, Switzerland) [41,42]. The Rancimat method was adopted to determine the effects of CA and TBHQ on the oxidation induction period (IP) of OA.

### 2.6. Assessment of the Oxidation Kinetics

To acquire the kinetic parameters, the kinetic rate constant (*k*) for lipid oxidation in OA was first calculated by using it as an inverse of IP (*k* = 1/*IP*, h^−1^) [43]. Ea (kJ/mol) and frequency factors (A, h^−1^) for oil oxidation could be obtained based on the Arrhenius equation:

ln (*k*) = ln (*A*) − E_a_/RT (3)

Typically, a linear curve could be constructed by using ln (*k*) as the ordinate, 1/T as the abscissa. As a result, the slope was −E_a_*/R*, and the intercept was ln (*A*). Therefore, the E_a_ value and frequency factors could be calculated. Moreover, according to activated complex theory (ACT), the activation enthalpies (ΔH) and entropies (ΔS) of oil oxidation were calculated using the equation [43]:

ln (*k*/T) = ln (*k*_B_/ℎ) + (ΔS/R) − (ΔH/RT), (4)

where *k*_B_ is the Boltzmann constant (1.380658 × 10^−23^ J/K), and *h* is the Planck’s constant (6.6260755 × 10^−34^ J s). Similarly, ΔH and ΔS were obtained from the slope and intercept of the equation.

For the analysis of OA stability, the Flynn–Wall–Ozawa and Kissinger–Akahira–Sunose methods were adopted [44,45]. The former method can be expressed as:

lg β = −0.4567 E/(RT_p_) + C1 (5)

And the latter one as:

lg β/T_p_^2^ = −0.4343 E/(RT_p_) + C2 (6)

where R is the thermodynamic constant (R ≈ 8.314 J/(mol·K)); β is the heating rates, K/min; T_p_ is the temperature of the sample at peak; C1 and C2 are constants.

### 2.7. ESR Spectroscopy Measurements

For the ESR measurements, PBN was dissolved in ethanol and then introduced to OA, and the concentration of PBN was 0.1 mg g^−1^. Next, the mixture was transferred to tubes (diameter 4 mm) for continuous heating at 180 °C. The samples were taken at a time interval of 0, 2 and 4 h, respectively. After the baseline correction, the ESR signals were detected by the ESR in Guangzhou, China (JES FA200 JEOL Electronics Co., LTD, Tokyo, Japan).

The parameters for ESR tests were described as follows: center field, 323.171 mT; sweep time, 1 min; microwave frequency, 9056.237 MHz; microwave power, 0.99800 MW; modulation frequency, 100 KHz.

### 2.8. Statistical Analysis

The tests for oxidation kinetics were performed three times, and the averaged data were employed to calculate the parameters. Statistical data analysis was carried out using the SPSS (version26.0, SPSS Inc., Chicago, IL, USA). Significance was defined at *p* < 0.05 unless otherwise mentioned.

## 3. Results

### 3.1. Mechanism of CA for Free Radicals Scavenge

#### 3.1.1. Geometric Configuration and Parameters of CA

CA is an antioxidant with an o-diphenol diterpene structure, the activity of which is originated from the two phenolic hydroxyl groups [46]. The optimized geometric CA configuration, at the B3LYP/6-31G (d,p) level, is proposed (Figure S1), and the selected main geometric parameters of CA chemical structure are listed in Table S1. The similar bond lengths of O(15)-H(52) and O(18)-H(47) are 0.975 Å and 0.968 Å, respectively, which are both longer than those of common phenolic hydroxyl (0.96 Å), indicating that the hydroxyl groups on the benzene ring are more active [47]. Meanwhile, a long-range **π**-conjugation can be formed via the oxygen atom of the hydroxyl group and the benzene ring, thus the electron density of the oxygen atom becomes weaker, which further reduces its binding ability to the adjacent hydrogen atom [30]. The hydroxyl hydrogen on the benzene ring of CA may easily be ionized, which is key for CA to exert its antioxidant functions [30,47].

Additionally, the O(15)-H(52) bond is 0.007 Å longer than the O(18)-H(47) bond, which can be ascribed to the lower attraction of the O(15) lone pair electron to H(52). Judging from the dihedral angle, the O(15)-H(52) and benzene ring are basically in the same plane, the H(52) atom deviates from the plane with 3.1°, while that of the O(18)-H(47) deviates from the benzene ring with 32.8°. As a consequence, the π-conjugation between O(15) and the benzene ring are more easily formed, which results in a longer bond length. In this respect, the O(15)-H(52) bond seems to be more active than the O(18)-H(47) bond. However, the force of intra-molecular hydrogen bonding, which can pose a great impact on the reaction activity, is not considered [48]. Therefore, the specific natural charge distribution and bond energy are more favorable to evaluate the group activity [30].

#### 3.1.2. Charge Distribution of CA Natural Bond Orbital (NBO)

Free radicals are more likely to attack atoms rich in electrons such as oxygen atoms [10,30]. The anti-oxidation process of CA toward OA can be regarded as stabilizing the free radicals. Since the phenolic hydroxyl groups of CA exhibit anti-oxidation activity, the more negative a charge that oxygen atoms possess, the easier the hydrogen atoms departure will be, due to the attack of free radicals. Thus, the most favorable site for free radical reaction can be the oxygen atom with the most negative charge.

NBO 3.0 was used to analyze the charge distribution of CA molecules at the level of B3LYP/6-31G (d,p), and the results are shown in Table S2. As observed, oxygen atoms possess the most negative charge. Amongst these, O(15) and O(18) belong to phenolic hydroxyl groups. The charge of O(18) is −0.73370 e, which is lower than that of O(15) (−0.70092 e). Hence, free radicals tend to more easily attack O(18), and the O(18)-H(47) is more active. Additionally, the charge of H (52) is nearly the same as that of H(47), which is consistent with the results of bond length.

#### 3.1.3. BDE of the Phenolic Hydroxyl in CA

BDE is regarded as the most convincing criterion for bond strength. BDE value of phenolic hydroxyl groups has widely been adopted to indicate their relative magnitude of antioxidant activity [49,50,51]. The lower BDE value the hydroxyl group is, the easier the bond will break when it reacts with free radicals [51]. Therefore, the BDE of phenolic hydroxyl groups in CA is directly related to their antioxidant activity towards OA.

To further verify the antioxidant activity of the two hydroxyl groups, we investigated their BDE with different methods. CA and its free radical energy were calculated at B3LYP/6-31G level (Table 1). Obviously, BDE of the O(15)-H (303.27 kJ/mol) was higher than that of the O(18)-H (295.63 kJ/mol), which revealed that the O(15)-H bond was fundamentally stronger than the O (18)-H bond. The above-mentioned bond length suggested a different result with BDE values, which was probably caused by ignoring the effect of intermolecular hydrogen bonding [52]. Overall, it was concluded that the most active site in CA was the O(18)-H hydroxyl group, followed by the O(15)-H hydroxyl group.

#### 3.1.4. The Distribution Pattern of Molecular Orbitals of CA

It is generally accepted that the HOMO and LUMO provide key information for the chemical reactions. A higher HOMO level is likely to donate electrons, and a lower LUMO level is likely to accept electrons [53]. As depicted in Figure S2, the red and green colors in the orbitals correspond to the positive and negative phases, respectively. The HOMO and LUMO orbitals mainly focus on the benzene ring, carboxyl and hydroxyl groups. Accordingly, C(12) adjacent to the benzene ring contributes to the HOMO orbital, and C(48) in the carboxyl group contributes to the LUMO orbital. Table S3 lists the orbital energies of CA that dissociate the hydrogen radical from O(15)-H and O(18)-H, where ΔE_(LUMO-HOMO)_ stands for the energy level difference, indicating the stability of the CA radical. The ΔE_(LUMO-HOMO)_ of CA is calculated to be 525.21 kJ/mol, which reveals that the CA radicals exhibit good stability, and the hydroxyl groups have strong activity.

### 3.2. Mechanism of the Hydrogen Extraction Reaction of α-C-H in OA

#### 3.2.1. Geometric Configuration and Parameters of OA

**Figure 1:**
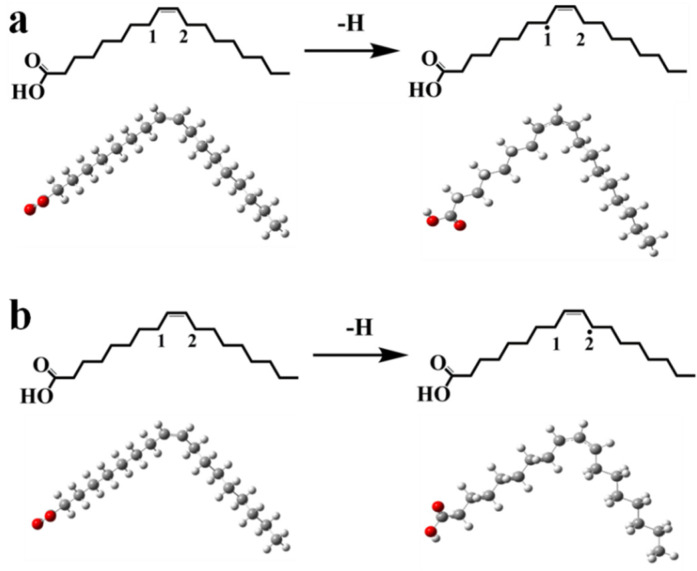
(**a**) Molecular structure of OA and OA radical on α-C (1#); (**b**) Molecular structure of OA and OA radical on α-C (2#).

Similarly, the optimized geometric configurations of OA and its radical, at the B3LYP/6-31G (d,p) level, were proposed and shown in Figure 1a,b, and the selected main geometric parameters are displayed in Table S4. After the hydrogen extraction reaction, the bond length of the other C-H bond decreases, which can be ascribed to the change in hybridization form, from sp^3^ to sp^2^.

#### 3.2.2. BDE of the α-C-H in OA

The BDE of the α-C-H was discussed to determine the active site of α-C-H in OA. The BDE values of the 1# α-C(8)-H and 2# α-C(11)-H were calculated to be 353.92 kJ/mol and 353.72 kJ/mol, respectively (Table 2). Both have nearly the same BDE value. However, the value of 2# α-C(11)-H was a little lower than that of 1# α-C(8)-H, which revealed that 2# α-H was more likely to go through the hydrogen extraction reaction. Both 1# α-C(8)-H and 2# α-C(11)-H were easily able to produce free radicals under a high temperature.

### 3.3. The Discussion of CA against the Oxidation of OA

#### 3.3.1. Theoretical Analysis of OA against CA Oxidation

After discussing the hydrogen extraction mechanism of OA and CA, the antioxidant reaction between CA and OA was explored. The above DFT theory results verified that the anti-oxidation reaction of CA involved a hydrogen atom transfer (HAT). The lower BDE of O(18)-H revealed that it was the favorable active site for HAT. Similarly, 2# α-C(11)-H exhibited a lower BDE than that of 1# α-C(8)-H, which suggested that the hydrogen abstraction preferentially occurred at α-C(11)-H under thermal oxidation. Thus, we chose the reaction of 2# α-C(11)-H and O(18)-H as the model to explain the anti-oxidation of CA towards OA.

**Figure 2:**
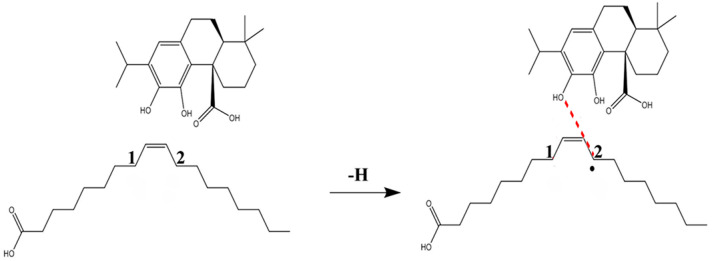
Reaction model of CA against the oxidation of OA (2#).

Based on the calculation (Figure 2), the thermal oxidation of OA first occurred at α-C(11)-H and produced the OA radical, followed by the α-C(8)-H. In the presence of CA, the H atoms at O(18)-H and O(18)-H were subsequently abstracted by OA radicals. The CA molecules finally formed a stable quinoid structure and the OA radical reaction was cut off. As such, the anti-oxidation process could be expressed as: ROO + R_1_(OH)_2_ → ROOH + R_1_ = O, where ROO· and R_1_(OH)_2_ represent OA radical and CA, respectively.

#### 3.3.2. Assessment of Thermal Stability

Upon different temperatures, a thermal oxidation tester was applied to measure the induction period (IP) of CA and TBHQ. The results are listed in Table S5, and the IP of OA, CA + OA, TBHQ + OA dramatically prolonged as temperature increased (*p* < 0.05). When the antioxidants were introduced, the IP of OA was significantly extended, and CA showed better performance than TBHQ (*p* < 0.05), which revealed that both CA and TBHQ could improve the stability of OA, with CA exerting a superior effect.

**Figure 3:**
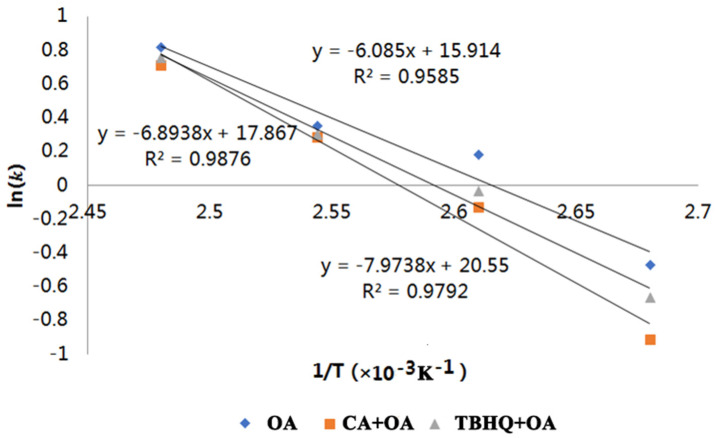
Calibration curves of ln (*k*) versus 1/T for OA, TBHQ + OA and CA + OA.

Based on the kinetic data given in Table S6, the calibration curves of ln (*k*) vs. 1/T were constructed in temperatures ranging from 100 to 130 °C. The relationship between ln (*k*) and 1/T can be described as linear regression equations for OA, CA + OA and TBHQ + OA, respectively (Figure 3). The obtained equations were ln (*k*) = −6.085/T + 15.914, R^2^ = 0.9585; ln (*k*) = −7.9738/T + 20.55, R^2^ = 0.9792; ln (*k*) = −6.8938/T + 17.867, R^2^ = 0.9876, and were in accordance with the Arrhenius equation at a temperature range between 100 and 130 °C. Subsequently, according to Equation (3), E_a_ of the reaction was calculated to be 50.59, 66.29 and 57.32 kJ/mol, respectively (Table 3). Previous research has shown that a higher E_a_ indicates a higher stability and lower oxidation rate [54]. Therefore, both CA and TBHQ improved the stability of OA, even though CA exerted a superior effect.

**Figure 4:**
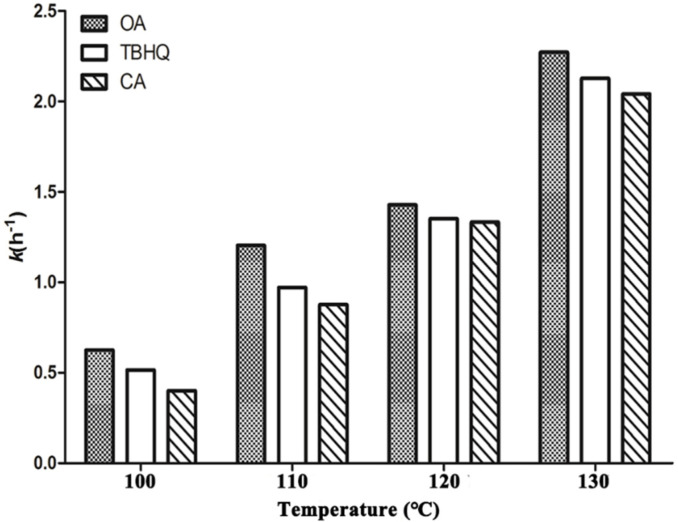
Kinetic rate constant (*k*) of the oxidation of OA, TBHQ + OA and CA + OA.

Furthermore, based on the oxidation kinetics of OA, the rate constant (*k*) can be used to indicate the oxidation stability of oils. As shown in Figure 4, the *k* values of OA, TBHQ + OA and CA + OA showed an uptrend with the increase in temperature, whereas a significant difference was observed after the addition of TBHQ and CA into OA (*p* < 0.05). Obviously, after introducing TBHQ and CA, the oxidation rate of OA decreased, wherein, CA exhibited a slightly higher antioxidant activity.

From the perspective of the activated complex theory, Δ*H* and Δ*S* exhibited a positive relationship with the thermal oxidation rate of oils [55]. Based on the data listed in Table S6, the plots of ln (*k*/T) versus 1/T were first established for OA, CA + OA and TBHQ + OA from 100 to 130 °C (Figure S1). The corresponding equations could be expressed as: y = −5.6975x + 8.9535, *R*^2^ = 0.9528; y = −6.5083x + 10.912, *R*^2^ = 0.9860; y = −7.5879x + 13.594, *R*^2^ = 0.9770. According to the equation of ln (*k*/T) = ln (*k*_B_/ℎ) + (ΔS/R) − (ΔH/RT), ΔH of OA, TBHQ + OA and CA + OA were calculated to be 47.37, 54.11 and 63.09 kJ/mol, and Δ*S* were −103.95, −87.66 and −65.36 J/mol K, respectively. The higher Δ*H* and Δ*S* of TBHQ + OA and CA + OA, than OA, revealed that both CA and TBHQ can effectively stabilize the oils under thermal oxidation. As CA was introduced, a more stable system could be obtained, and this result is in accordance with those previously presented.

#### 3.3.3. The Analysis of OA Stability by DSC

In addition, differential scanning calorimetry (DSC) was carried out to explore OA stability before and after the addition of CA and TBHQ, and the data were displayed in Table S7. Based on the Flynn–Wall–Ozawa and Kissinger–Akahira–Sunose methods [44,45], the regression equations of lgβ versus 1/T were fitted for OA, OA + CA and OA + TBHQ (Table S8). For the Flynn–Wall–Ozawa method, the equations could be expressed as: y = −2.7636x + 6.2864, *R*^2^ = 0.9965; y = −2.8881x + 6.454, *R*^2^ = 0.9883; y = −2.8294x + 6.3863, *R*^2^ = 0.9960, and the calculated *E_a_* values were 50.31, 52.58 and 51.51 kJ/mol, respectively. The equations obtained by the Kissinger-Akahira-Sunose method, could be described as: y = −2.3126x − 0.0147, *R*^2^ = 0.9960; y = −2.4308x + 0.1407, *R*^2^ = 0.9853; y = −2.3751x + 0.0789, *R*^2^ = 0.9952; and the E_a_ values were 44.27, 46.53 and 45.47 kJ/mol, respectively. It was apparent that both models produced the same order of *E_a_* values, that is, CA + OA > TBHQ + OA > OA, which was in accordance with the results of ln (*k*) versus 1/T equations. Overall, the results revealed that both CA and TBHQ generated antioxidant activity towards OA, and the oxidation stability follows the order of CA + OA > TBHQ + OA > OA.

#### 3.3.4. Free Radicals Scavenging Activity and ESR Analysis of OA Stability

As demonstrated, the thermal oxidation of oils underwent a free radical reaction, and CA and TBHQ could inhibit the process by scavenging the free radicals. Moreover, as listed in Table S9, the scavenging ability of CA on free radial DPPH^•^ (10.50 μg/mL) was stronger than that of TBHQ (15.50 μg/mL), while the scavenging ability on free radical ABTS^•+^ (17.11 μg/mL) was weaker than that of TBHQ (13.14 μg/mL). Hence, the free radical scavenging activity of CA predicted by theoretical data was basically consistent with the experimental results, and CA can be considered as a substitute for TBHQ. Additionally, from the view of the structure, TBHQ is a diphenol antioxidant with two hydrogen atoms, which can easily form quinone. With the diphenol diterpenoid structure, CA is similar to TBHQ and also has two active hydroxyl groups. Thus, the antioxidant effect of CA is almost the same as that of TBHQ, indicating that CA is an effective natural antioxidant.

**Figure 5:**
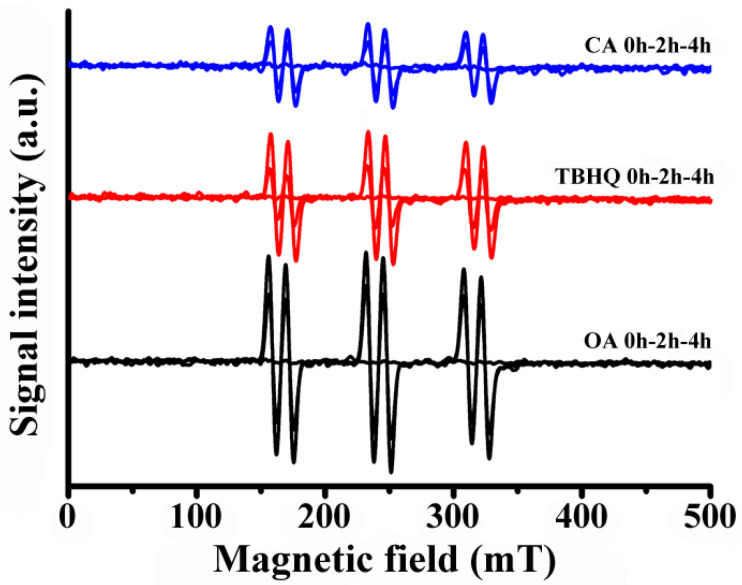
ESR spectra of PBN-free radical adducts over OA with and without CA and TBHQ under thermal oxidation for 0 h, 2 h, 4 h, respectively.

To further verify this effect, ESR tests were carried out to detect the free radicals in the OA oxidation process. The ESR spectrum of OA oxidation before and after adding CA and TBHQ are shown in Figure 5. When the samples were incubated with PBN under 180 °C for a certain period, they exhibited six featured peaks for PBN-free radical adducts, which indicated that free radicals were produced under applied thermal conditions. Additionally, a stronger intensity could be observed for longer incubation periods (2 h, 4 h), revealing that free radicals were increased by increasing the incubation time. Additionally, OA exhibited stronger intensity compared with that of OA + CA and OA + TBHQ. These data suggested that CA and TBHQ could inhibit the generation of free radicals throughout OA oxidation. Additionally, the signal that originated from OA + TBHQ system was higher than that of the OA + CA system, suggesting that CA was more active than TBHQ in inhibiting the thermal oxidation of oils.

## 4. Conclusions

The antioxidant mechanism of CA is primarily based on the hydrogen atom transfer (HAT) reaction. The antioxidant activity of CA originated from the two phenolic hydroxyl groups, and O(18)-H showed higher activity than O(15)-H with lower BDE. After introducing CA and TBHQ in OA oxidation reaction system, the oxidation rate greatly reduced, and CA showed better antioxidant performance. The kinetic parameters such as Δ*H*, Δ*S* and *Ea* were calculated to demonstrate the activity of CA and TBHQ. In the OA oxidation process, the abstract of hydrogen first occurred at α-C(11)-H and produced the OA radical, followed by the α-C(8)-H. The CA molecules donated the active hydrogen at O(18)-H and O(15)-H, and finally formed a stable quinoid structure and stopped the OA radical reaction. Overall, CA is an excellent antioxidant that can effectively inhibit thermal oxidation of OA, with the potential to be a substitute for TBHQ in foods.

## Figures and Tables

**Table 1:** BDE of CA molecular and its free radicals (—not given).

Free Radicals	Energy (kJ/mol)	BDE (kJ/mol)
CA	−2,836,114.23	—
CA-(15)O	−2,834,498.68	303.27
CA-(18)O	−2,834,506.32	295.63
H	−1312.28	—

**Table 2:** The values of E_sr_, E_hr_ and E_s_ and BDE.

Molecules	1# α-C(8)-H	2# α-C(11)-H
OA (E_s_, hartree)	−856.390	−856.390
α-H (E_hr_, hartree)	−0.498	−0.498
OA radicals (E_sr_, hartree)	−855.757199	−855.757275
α-C-H (BDE, kJ/mol)	353.92	353.72

**Table 3:** Arrhenius parameters, ΔH, and ΔS of OA, TBHQ + OA and CA + OA.

	ln (*k*) = ln (*A*) − E_a_/RT (3)		ln (*k*/T) = ln (*k_B_*/*h*) + ΔS/R − ΔH/RT (4)			
	Ea (kJ/mol)	R^2^	ln (kB/h)	ΔH (kJ/mol)	ΔS (J/molK)	R^2^
OA	50.59	0.9585	21.46	47.37	−103.95	0.9528
TBHQ + OA	57.32	0.9876	21.46	54.11	−87.66	0.9860
CA + OA	66.29	0.9792	21.46	63.09	−65.36	0.9770

## Data Availability

No new data were created or analyzed in this study. Data sharing is not applicable to this article.

## Supplementary Materials

The following are available online at https://www.mdpi.com/article/10.3390/foods10102279/s1, Figure S1: Optimized configuration of CA (red: O atom; dark grey: C atom; light grey: H atom); Figure S2: The frontier molecular orbital distributions for CA; Table S1: The main molecular structure parameters of CA (—not given); Table S2: Atom charge population analysis of phenolic hydroxyl group in CA; Table S3: The values of EHOMO, ELUMO, and ΔE(LUMO-HOMO) of CA radicals; Table S4: The main molecular structure parameters of OA (—not given); Table S5: Induction period of different components at different temperatures; Table S6: Kinetics data of OA oxidation before and after adding CA and TBHQ; Table S7: Tp values at different heating rates (βi) for OA oxidation before and after adding CA and TBHQ; Table S8: Regression equations and Ea for OA, TBHQ + OA and CA + OA; Table S9: Antioxidant capacity of CA and TBHQ (IC50).
