# Supplementary material for: A Deep Insight in the Antioxidant Property of Carnosic Acid: From Computational Study to Experimental Analysis
Source: Foods. 2021 Sep 26;10(10):2279. doi: 10.3390/foods10102279 (PMC8534978; doi:10.3390/foods10102279)
Supplement: Supplementary file 1 [file foods-10-02279-s001.zip › foods-1391578-supplementary.pdf]

A deep insight in the antioxidant property of carnosic acid: From computational study to experimental analysis

Jing Wei[1,3]*, Qian Liang*[1]*, Yuxin Guo*[1]*, Weimin Zhang*[*,1,3], *Long Wu*[1,2]

[1] College of Food Sciences & Engineering, Hainan University, Engineering Research Center of Utilization of Tropical Polysaccharide Resources (Ministry of Education), 58 People Road, Haikou 570228, PR China.; longquan.good@163.com; zhwm1979@163.com

[2] Hubei Key Laboratory of Industrial Microbiology, Key Laboratory of Fermentation Engineering (Ministry of Education), Hubei University of Technology, Wuhan, Hubei 430068, P.R. China; longquan.good@163.com

[3] Key Laboratory of Tropical Fruits and Vegetables Quality and Safety for State Market Regulation. Hainan Institute for Food Control,285 Nanhai Road, Haikou 570314, PR China.; weijing0128@126.com; zhwm1979@163.com

College of Food Science and Engineering,

Hainan University

58 Renmin Ave, Meilan District, Haikou City, Hainan Prov. 570228, PR China

Tel:+0898-66193581

Fax:+0898-66193581

*Corresponding Author E-mail

Weimin Zhang: zhwm1979@163.com

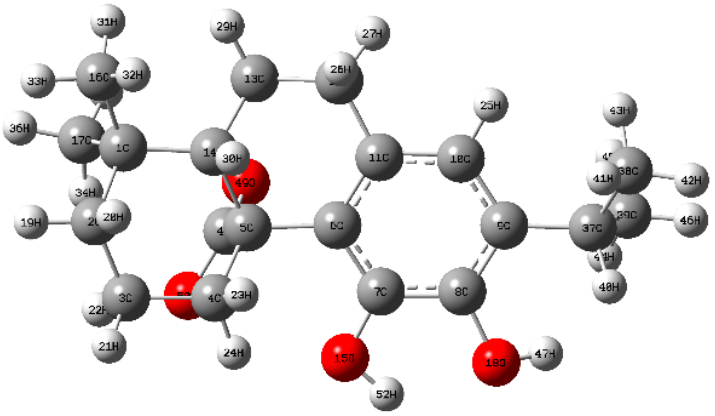

Figure S1. Optimized configuration of CA (red：O atom；dark grey：C atom；light grey： H atom).

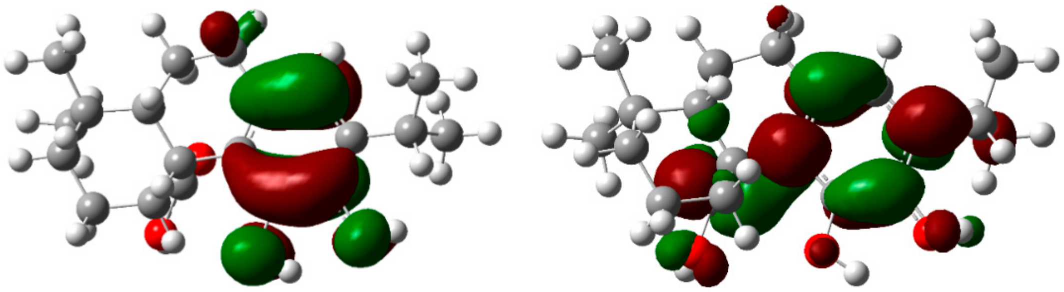

HOMO **LUMO**

Figure S2. The frontier molecular orbital distributions for CA (red spheres represent O atoms; the brown and green colors in the orbitals are corresponded to the positive and negative phase).

Table S1. The main molecular structure parameters of CA (—not given).

Bond length (Å)		Bond angle (°)		Dihedral angle (°)	
C(7)-O(15)	1.368	C(6)-C(7)-O(15)	120.948	C(6)-C(7)-O(15)-H(52)	176.906
C(8)-O(18)	1.393	C(8)-C(7)-O(15)	117.688	C(7)-C(8)-O(18)-H(47)	147.181
C(48)-O(49)	1.214	C(7)-C(8)-O(18)	114.139	C(5)-C(6)-C(11)-C(12)	−1.7385
C(48)-O(50)	1.357	C(9)-C(8)-O(18)	123.926	C(6)-C(11)-C(12)-C(13)	−16.631
O(15)-H(52)	0.975	C(7)-O(15)-H(52)	106.041	C(11)-C(6)-C(5)-C(14)	−13.558
O(18)-H(47)	0.968	C(8)-O(18)-H(47)	109.549	—	—
O(50)-H(51)	0.976	—	—	—	—

Table S2. Atom charge population analysis of phenolic hydroxyl group in CA.

Atom	Natural charge (e)	Atom	Natural charge (e)	Atom	Natural charge (e)
C(6)	–0.06656	O(15)	–0.70092	H(25)	0.22997
C(7)	0.31058	O(18)	–0.73370	H(47)	0.50072
C(8)	0.25015	O(49)	–0.61439	H(51)	0.49577
C(9)	–0.07554	O(50)	–0.70375	H(52)	0.50555

Table S3. The values of E_{HOMO}, E_{LUMO}, and $\Delta E_{(LUMO\text{-}HOMO)}$ of CA radicals.

No.	Orbital	E（kJ/mol）	No.	Orbital	E（kJ/mol）
95	LUMO+4	1858.3973	90	HOMO	–530.35104
94	LUMO+3	119.434	89	HOMO-1	–585.56531
93	LUMO+2	77.347236	88	HOMO-2	–676.90646
92	LUMO+1	43.189478	87	HOMO-3	–724.48053
91	LUMO	–5.1459804	86	HOMO-4	–729.12766
	$\Delta E_{(LUMO\text{-}HOMO)}$ (kJ/mol)			525.20506	

The first, second, third, and forth occupied (unoccupied) molecular orbitals below (above) the HOMO (LUMO) have been designated as HOMO-1 (LUMO+1), HOMO-2 (LUMO+2), HOMO-3 (LUMO+3) and HOMO-4 (LUMO+4), respectively.

Table S4. The main molecular structure parameters of OA (—not given).

Molecule	1# α-C(8)-H bond length/Å	2# α-C(11)-H bond length/Å
Reactant C_1	1.101	1.101
	1.095	1.095
Reactant C_2	1.100	1.100
	1.095	1.095
Product C_1	1.089	1.103
	—	1.092
Product C_2	1.101	1.089
	1.093	—

Table S5. Induction period of different components at different temperatures.

Temperature (°C)	OA (h)	CA + OA (h)	TBHQ + OA (h)
100	1.6	2.49	1.94
110	0.83	1.14	1.03
120	0.70	0.75	0.74
130	0.44	0.49	0.47

Table S6. Kinetics data of OA oxidation before and after adding CA and TBHQ.

Temperature (°C)	100	110	120	130
T (K)	373.15	383.15	393.15	403.15
1/T（$\times 10^{-3}$ K^{-1})	2.68	2.61	2.544	2.48
OA (*IP*)	1.60±0.02b	0.83±0.01b	0.70±0.01b	0.44±0.01b
K (*1/IP*)	0.625	1.204819	1.428571	2.272727
lnk	–0.7	0.18633	0.356675	0.820981
k/T	0.001675	0.003145	0.003634	0.005637
ln (*k/T*)	–6.39194	–5.76194	–5.61742	–5.1784
CA + OA (*IP*)	2.49±0.02a	1.14±0.03a	0.75±0.01a	0.49±0.02a
K (*1/IP*)	0.401606	0.877193	1.333333	2.040816
lnk	–0.91228	–0.13103	0.287682	0.71335
k/T	0.001076	0.002289	0.003391	0.005062
ln (*k/T*)	–6.8345	–6.07964	–5.68663	–5.28599
TBHQ+OA (*IP)*	1.94±0.03a	1.03±0.02a	0.74±0.01a	0.47±0.02a
K (*1/IP*)	0.515464	0.970874	1.351351	2.12766
lnk	–0.66269	–0.02956	0.301105	0.755023
k/T	0.001381	0.002534	0.003437	0.005278
ln (*k/T*)	–6.58495	–5.97796	–5.67316	–5.24421

Table S7. T_p values at different heating rates (β_i) for OA oxidation before and after adding CA and TBHQ.

B (°C/min)	5	10	15	20
lgβ	0.70	1.00	1.18	1.30
OA (T_p) (K)	494.05	524.982	539.881	553.981
1/T ($\times 10^{-3}$) (K^{-1})	2.024087	1.904827	1.85226	1.805116
lg (β/T^2)	–4.6885718	–4.44029	–4.2885	–4.18596
TBHQ (T_p) (K)	498.15	525.176	541.15	558.15
1/T ($\times 10^{-3}$) (K^{-1})	2.007427	1.904124	1.847916	1.791633
lg (β/T^2)	–4.69575	–4.44061	–4.29054	–4.19247
CA (T_p) (K)	502.75	530.077	543.47	563.157
1/T ($\times 10^{-3}$) (K^{-1})	1.98906	1.886518	1.840028	1.775704
lg (β/T)	–4.70373	–4.44868	–4.29426	–4.20023

Table S8. Regression equations and E_a for OA, TBHQ+OA and CA+OA.

	**Flynn-Wall-Ozawa method**			**Kissinger-Akahira-Sunose method**		
	Equation	R^2	E_a (kJ/mol)	Equation	R^2	E_a（kJ/mol）
OA	y = –2.7636x + 6.2864	0.9965	50.31	y = –2.3126x – 0.0147	0.9960	44.27
OA + TBHQ	y = –2.8294x + 6.3863	0.9960	51.51	y = –2.3751x + 0.0789	0.9952	45.47
OA + CA	y = –2.8881x + 6.454	0.9883	52.58	y = –2.4308x + 0.1407	0.9853	46.53

Table S9. Antioxidant capacity of CA and TBHQ（IC_{50}）.

Antioxidants	$DPPH^{\bullet}$ (μg/mL)	$ABTS^{\bullet+}$ (μg/mL)
CA	10.50	27.11
TBHQ	15.50	13.14
